# Prediction and analysis of metagenomic operons via MetaRon: a pipeline for prediction of *Meta*genome and whole-genome ope*Ron*s

**DOI:** 10.1186/s12864-020-07357-5

**Published:** 2021-01-19

**Authors:** Syed Shujaat Ali Zaidi, Masood Ur Rehman Kayani, Xuegong Zhang, Younan Ouyang, Imran Haider Shamsi

**Affiliations:** 1 Bioinformatics Division, Beijing National Research Institute for Information Science and Technology (BNRIST), Department of Automation, Tsinghua University, Beijing, 100084 People’s Republic of China; 2 Bioscience Department, COMSATS Institute of Information Technology, Islamabad, 44000 Pakistan; 3 Center for Innovation in Brain Science, University of Arizona, Tucson, 85719 USA; 4 Center for Microbiota and Immunological Diseases, Shanghai General Hospital, Shanghai Institute of Immunology, Shanghai Jiao Tong University, School of Medicine, Shanghai, 2000025 People’s Republic of China; 5 China National Rice Research Institute (CNRRI), 28 Shuidaosuo rd, Fuyang, Hangzhou, 311400 People’s Republic of China; 6 Department of Agronomy, College of Agriculture and Biotechnology, Key Laboratory of Crop Germplasm Resource, Zhejiang University, Hangzhou, 310058 People’s Republic of China

**Keywords:** *Escherichia coli*, Metagenomic, Operon prediction, Secondary metabolites, Microbiome

## Abstract

**Background:**

Efficient regulation of bacterial genes in response to the environmental stimulus results in unique gene clusters known as operons. Lack of complete operonic reference and functional information makes the prediction of metagenomic operons a challenging task; thus, opening new perspectives on the interpretation of the host-microbe interactions.

**Results:**

In this work, we identified whole-genome and metagenomic operons via MetaRon (Metagenome and whole-genome opeRon prediction pipeline). MetaRon identifies operons without any experimental or functional information. MetaRon was implemented on datasets with different levels of complexity and information. Starting from its application on whole-genome to simulated mixture of three whole-genomes (*E. coli* MG1655, *Mycobacterium tuberculosis* H37Rv and *Bacillus subtilis* str. 16), *E. coli* c20 draft genome extracted from chicken gut and finally on 145 whole-metagenome data samples from human gut. *MetaRon* consistently achieved high operon prediction sensitivity, specificity and accuracy across *E. coli* whole-genome (97.8, 94.1 and 92.4%), simulated genome (93.7, 75.5 and 88.1%) and *E. coli* c20 (87, 91 and 88%,), respectively. Finally, we identified 1,232,407 unique operons from 145 paired-end human gut metagenome samples. We also report strong association of *type 2 diabetes with* Maltose phosphorylase (K00691), 3-deoxy-D-glycero-D-galacto-nononate 9-phosphate synthase (K21279) and an uncharacterized protein (K07101).

**Conclusion:**

With *MetaRon,* we were able to remove two notable limitations of existing whole-genome operon prediction methods: (1) generalizability (ability to predict operons in unrelated bacterial genomes), and (2) whole-genome and metagenomic data management. We also demonstrate the use of operons as a subset to represent the trends of secondary metabolites in whole-metagenome data and the role of secondary metabolites in the occurrence of disease condition. Using operonic data from metagenome to study secondary metabolic trends will significantly reduce the data volume to more precise data. Furthermore, the identification of metabolic pathways associated with the occurrence of *type 2 diabetes* (T2D) also presents another dimension of analyzing the human gut metagenome. Presumably, this study is the first organized effort to predict metagenomic operons and perform a detailed analysis in association with a disease, in this case *type 2 diabetes*. The application of MetaRon to metagenomic data at diverse scale will be beneficial to understand the gene regulation and therapeutic metagenomics.

## Background

Bacteria present in diverse environments adaptively transcribe to flourish in dynamic conditions [1–3]. They survive in such conditions through the organization and clustering of two or more genes into a regulatory unit known as an operon [4–9]. Operons play an important role in the evolution of new proteins, enzymes, and pathways; and are vital for the production of natural products - many of which have therapeutic importance [10–14].

Contemporary studies have abundantly identified natural products helpful in treatment/prevention of cancer, diabetes, and lowering cholesterol [15]. Many of these products have operonic origins [16, 17]. Metagenomic access to novel environments also underscored the potential of operons in identification and functionality of uncultured microbial communities (taxonomic profiling, secondary metabolites, drug discovery and many others) [17–25].

Most whole-genome operon prediction methods depend on experimental or functional information in combination with computational parameters [11]; however, experimental/functional information about operons is absent in metagenomic data. Few whole-metagenome studies focused on exploring the operonic aspect of the environment including secondary metabolites and differentially abundant pathways of operonic origin [26–30].

Metagenomic operon prediction thus remains an understudied plane. Operons aiding microbial survival are crucial in understanding the gene regulation, identification of new pathways and novel products in diverse environmental settings. Experimental identification of metagenomic operons is an intensive and challenging process due to everchanging formulation of operons with respect to environmental stimulus. Therefore, computational operon prediction is an efficient way to identify operons. Metagenomic data contains a cumulative mixture of environmental DNA from millions of cultivable and uncultivable microbes. However, to our knowledge, there is no computational pipeline dedicated to predicting metagenomic operons without any functional information. Considering the importance of operons in bacterial survival, the development of a convenient automated solution independent of functional and experimental information is indispensable.

To overcome the limitations mentioned above, we present *MetaRon*, a *Meta*genomic and whole-genome ope*ron* prediction pipeline for shotgun sequencing data. *MetaRon* is a user-friendly pipeline that performs necessary downstream data processing (de novo assembly, gene prediction, de novo promoter prediction and proximon prediction), before identifying the operons from the metagenomic sample. In case of availability of pre-assembled metagenome and genes, *MetaRon* also predicts the operons, directly from scaftigs. The pipeline performs operon prediction with high sensitivity based on co-directionality, intergenic distance, and presence/absence of a promoter upstream and downstream of a gene. This pipeline will be beneficial in studying microbial gene regulation, pathways and secondary metabolites.

## Methods

### Implementation

*MetaRon* is developed and implemented in python 3.7. One successful run of *MetaRon* produces several tab delimited and fasta files containing different levels of information. This information will be used for further analysis of metagenomic operons.

#### Data input

**Fig. 1:**
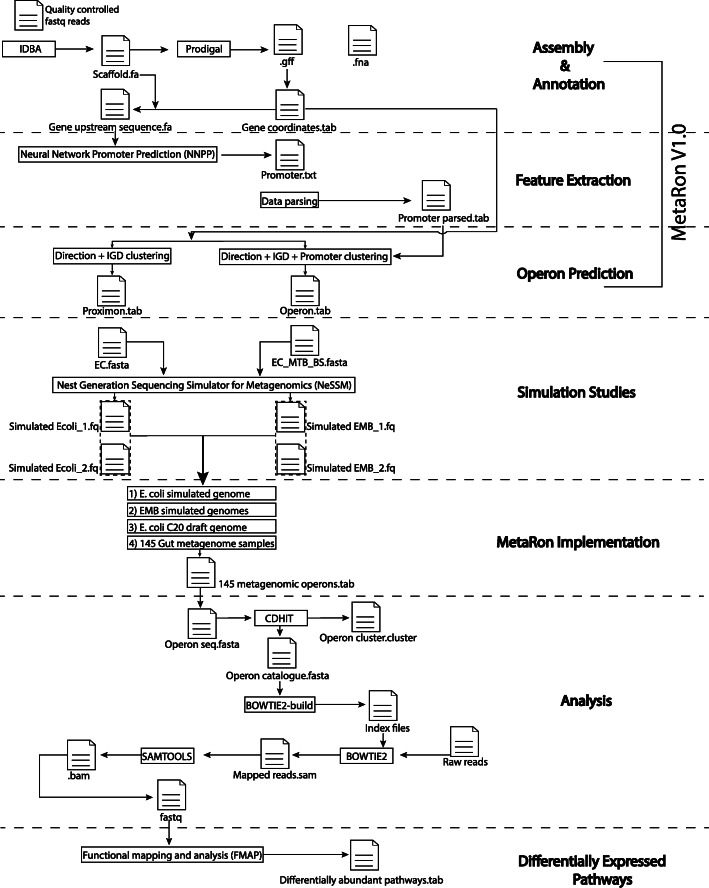
A detailed workflow demonstrating the prediction and analysis of metagenomic operons via MetaRon

*MetaRon* executes two type of workflows depending on the user input. The process parameter “**ago**” (Assembly, Gene prediction and Operon prediction) performs downstream data processing using trimmed and quality controlled metagenomic or whole-genome shotgun sequencing reads (Fig. 1). This includes de novo assembly via IDBA [31] and prediction of genes via Prodigal [32]. Alternatively, the user can also input assembled metagenomic scaftigs and gene prediction file (.gff), by specifying the process parameter “**op**” (Operon Prediction). The selection of “op” process will skip the downstream data processing steps directing the program to perform operon prediction only, as shown in Fig. 1. At this point it is important to mention that *MetaRon* only accepts gene prediction files produced by Prodigal and MetaGeneMark. The program requires the user to specify the gene prediction tool used to identify genes.

#### Feature extraction

Once *MetaRon* reaches the point where it contains de novo assembled scaftigs and gene prediction file, either via process “ago” or “op”, the process of operon prediction is the same (Fig. 1).

1. The ***data_extraction()*** module mines the gene prediction file (.gff file) and parses information including gene name, gene start and end coordinates, gene direction, and scaftig name into a matrix.
2. Next, the module ***seq_info()*** creates a dictionary of the scaftig name and scaftig length.
3. The output matrices of ***data_extraction()*** and ***seq_info()*** are used to calculate the upstream and downstream intergenic regions of the genes via ***upstream_coordinates_extraction()*** and ***downstream_coordinates_extraction()*** modules, respectively.
4. Subsequently, ***UPS_DSS_Slicing()*** trims down the upstream and downstream coordinates longer than 700 bp to 700 bp. Also, if the upstream or downstream region of a gene is shorter than 15 bp, it will be assigned a tag “short_ups” and “short_dss”, respectively (Fig. 1). These sequences will be ignored in forthcoming steps since signatures for promoter or terminator only appears on/after 15 bp.
5. The consequent step is the extraction of upstream and downstream sequence based on the trimmed coordinates (<= 700 bp). Module ***getsource()*** extracts scaftig information from the scaftig file in the form of a dictionary ***(d)***.
6. The ***getgenstring_ups()****,* and ***getgenstring_dss()*** modules extracts fasta sequence from the dictionary ***(d)*** using the trimmed upstream and downstream coordinates. The upstream fasta sequence is then used to predict the promoters.

The above-mentioned steps will produce a list of genes with trimmed coordinates and their sequences (upstream and downstream sequences). These coordinates will be used to identify the proximons from the metagenomic data.

#### Proximon identification

*MetaRon* will now identify the co-directional gene clusters and calculate the intergenic distance (IGD) (Eq. 1) between the genes in the clusters through ***IGD_calc()***. Intergenic distance is by far the most common parameter used for the prediction of operons in whole-genomes [6, 12, 14, 33–35]. The intergenic distance (IGD) between two genes is calculated as:

1 $$ \boldsymbol{IGD}\ \left(\boldsymbol{G}\mathbf{1},\boldsymbol{G}\mathbf{2}\right)=\left(\boldsymbol{start}\left(\boldsymbol{G}\mathbf{2}\right)-\boldsymbol{end}\left(\boldsymbol{G}\mathbf{1}\right)\right)+\mathbf{1} $$

Where, **G1** and **G2** are two adjacent co-directional genes, start *(G2)* refers to the beginning position of second gene in the pair on the genome, while end *(G1)* refers to the last nucleotide position of the first gene.

Various operon prediction methods use different range of intergenic distance to identify operons. Based on a thorough review of literature, *MetaRon* defines a flexible (< 601 bp) maximum threshold for Intergenic distance, which was also used by fuzzy genetic algorithm to identify operons [36]. This threshold is defined as a stretchy parameter due to extremely personalized and diverse definition of IGD in various bacterial species [11]. Furthermore, there is no universal threshold for intergenic distance defined for microbes. For metagenomic data, where there are millions of unrelated microbes, a flexible range of intergenic distance will ensure engulfing of all operonic genes in the gene cluster. However, a flexible threshold for intergenic distance will also allow the addition of many non-operonic genes into the cluster. These non-operonic genes will be removed later. Since these gene clusters are based on proximal genes and co-directionality, they are known as proximons.

The proximons gene clusters also struggle to accurately identify the transcription unit boundary (TUB). Hence, there is a need to accurately identify the transcription unit boundary within each proximons cluster, that will not only remove the non-operonic genes from the cluster but also delimit consecutive operons that were identified as one proximon. These delimited gene clusters with TUB defined will be called operons.

#### Operon prediction

The module ***promoter_prediction()*** integrates Neural Network Promoter Prediction 2.0 (NNPP)*,* to predict the upstream promoter for each of the genes in the co-directional closely packed gene clusters [37]. The output is organized into a matrix via ***Promoter_file_parse()***. The promoter prediction matrix will be integrated with proximon table and TUBs will be defined, using ***Prom_IGD_Clustering()***.

At this moment, an operon is defined as a cluster of two or more co-directional and closely packed genes with a promoter upstream of the first gene. As the structure of operon indicates, an operon starts with a promoter and ends with a terminator, sandwiching multiple genes within. However, the presence of a promoter downstream of the last gene of the operonic cluster could also signify the end of an operon and start of a new TUB for gene (*i + 1*)*.* Therefore, to redefine, an operon is a gene cluster delimited by an upstream and downstream promoter indicating the start and end of the operon, respectively.

Unlike *Prom_IGD_Clustering(),* where co-directionality, IGD and presence of promoter were considered to define an operon, the module ***Promoter_clustering()*** predicts the operons without considering intergenic distance at all. The pipeline compiles and exports the proximon pairs, and operons in tab-delimited files. Moreover, transitional information such as gene prediction file, upstream and downstream coordinates and fasta files are also available to the user for further analysis (Fig. 1).

*MetaRon* was implemented on whole-genome, simulated genomes, draft genome and whole-metagenomes, thus demonstrating its performance consistency at different levels of data complexity. The reason was to test the pipeline with different levels of data complexity, both in terms of diversity, information and data format such as, whole-genome or multiple scaftigs. For each of the data input, operons were identified, however, only the metagenomic data was analyzed in detail for its association with *type 2 diabetes* (T2D).

### Data analysis

After identification of operons from 145 human gut microbiome samples. We carried out a comprehensive analysis of metagenomic operons, which mainly includes a comparative analysis of biosynthetic gene clusters (BGCs) from operonic origin and whole-scaftig, in addition to the differential pathway analysis from operonic gene clusters.

### Secondary metabolite identification

Secondary metabolites were identified separately from operonic and complete scaftig sequences using antiSMASH (v3.0) (antibiotic and secondary metabolites analysis shell) with default parameters [38]. The operonic sequences were available as the final output file produced by *MetaRon*, while scaftigs were available as the output of de novo assembly in the data processing step of *MetaRon*. A comparative approach was devised to observe the abundance of secondary metabolites in operonic sequences as well as scaftigs for control and *type 2 diabetic* group of individuals.

### Functional mapping and pathway analysis

A mapping activity was being carried out all this while where raw metagenomic reads from all 145 samples were individually mapped to the operonic sequences using BOWTIE2 [39]. The resulting 145 sam files were processed using SAMtools [40]. This includes the conversion of sam files to bam and finally to fastq file format. The raw metagenomic reads aligned to the operonic sequences were then analyzed for differential pathways via a standalone pipeline for functional analysis FMAP (Functional Mapping and Analysis Pipeline) [41]. Mapping hits that qualified through the default FMAP settings (sequence identity = > 80%, e-value = > 1e-10) mapped to the KEGG Orthology (KO) database [42, 43]. The mapped reads were then normalized to the total number of paired-end reads. The normalized abundance for each sample was calculated as the number of reads aligned to a gene divided by total read count, followed by a summation of all the genes in the pathway. FMAP pipeline also mapped of raw metagenomic reads to the UniRef100 [44] reference database using DIAMOND [45] and estimated the gene abundance to identify the differentially abundant pathways and modules.

## Results and discussion

Most of the previous whole-genome operon prediction methods depend highly on experimental and functional information such as microarray data, metabolic pathways, Gene Ontology (GO), and Cluster of Orthologous Groups (COGs). Unavailability of such information in most instances of metagenomic data makes metagenonmic operon prediction a tricky task [34, 46–52]. We addressed these limitations via *MetaRon*, by accurately predicting metagenomic operons independent of functional or experimental information. Although, Vey (2013) demonstrated that metagenomic operons can be identified without any functional or experimental information [53], handling of huge metagenomic data manually is often tedious and prone to errors. Therefore, MetaRon presents an automated, improved and universal solution towards the prediction of operons in whole-genome and metagenome shotgun sequencing data.

### Data sources

MetaRon utilizes multiple data types and sources. Raw reads of Escherichai coli K-12 MG1655 (SRP029211), Whole-genome of *Escherichia coli* MG1655 (NC_000913.3), Bacillus subtilis 168 (NC_000964), *Mycobacterium tuberculosis* H37Rv (NC_000962) and *Escherichia coli* C20 draft genome (NGBR00000000.1) were downloaded from the NCBI, Genome database. Human gut metagenomic shotgun sequencing reads from 145 Chinese individuals (Table 1), were retrieved from the European Bioinformatics Institute (SRP008047) [54].

**Table 1:** Number of samples belonging to each group of individuals

Category	Count
**Disease Lean Female (DLF)**	12
**Disease Lean Male (DLM)**	26
**Disease Obese Female (DOM)**	13
**Disease Obese Male (DOM)**	20
**Normal Lean Female (NLF)**	13
**Normal Lean Male (NLM)**	24
**Normal Obese Female (NOF)**	13
**Normal Obese Male (NOM)**	24

### MetaRon application

#### Whole-genome

**Fig. 2:**
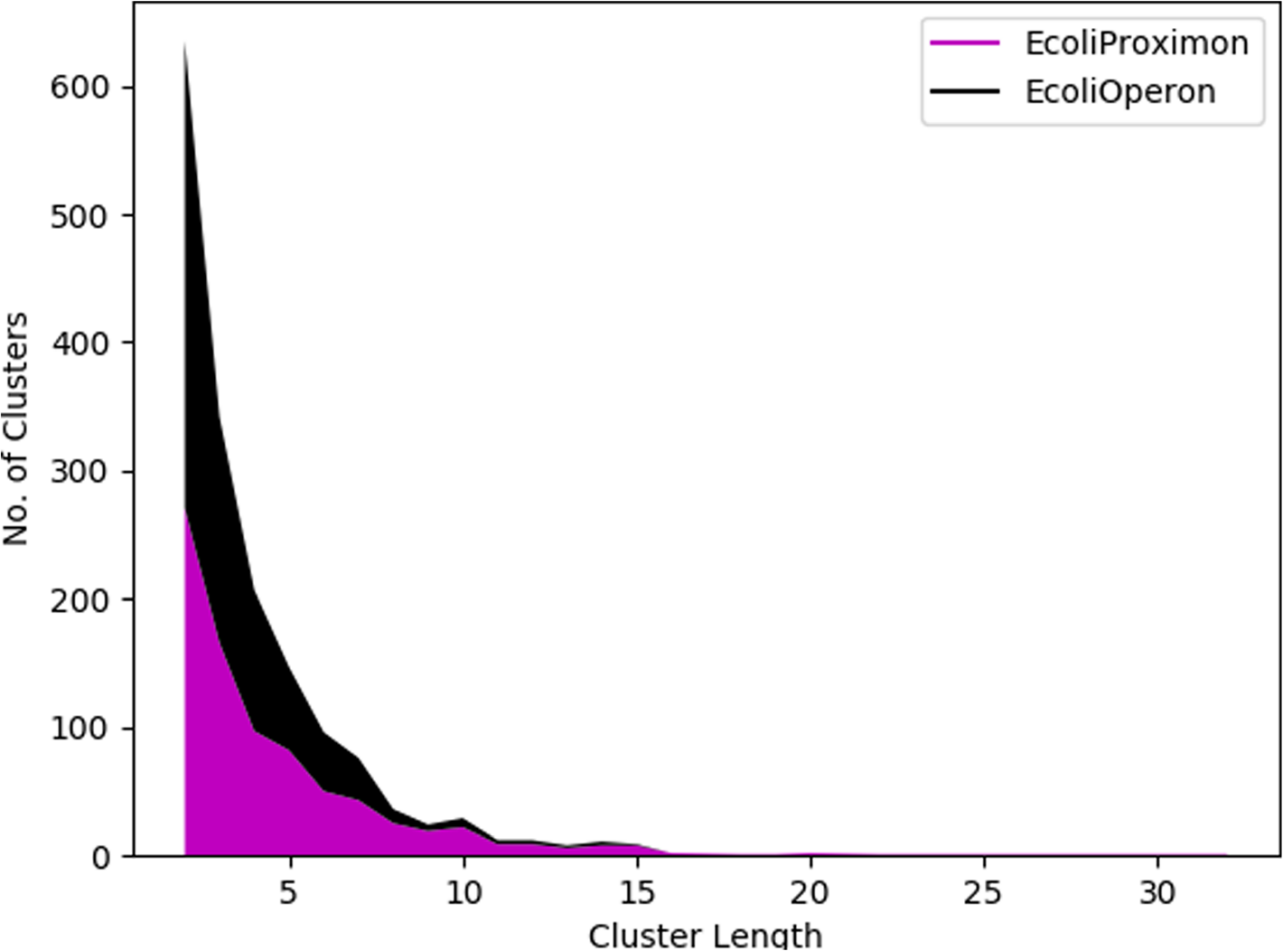
The distribution of operonic and proximonic gene clusters by length

*E. coli K-12 MG1655* is considered as the gold standard in terms of operons, since it contains the most complete set of operonic information validated experimentally. That is the reason, most of the operon prediction methods were designed and tested on it. We also implemented *MetaRon* on illumine HiSeq reads of *E. coli K-12 MG1655* as the first run. 82 scaftigs were assembled by *MetaRon* via IDBA [55]. Scaftigs with length less than or equal to 500 bp were removed. The remaining scaftigs resulted in 4227 genes, predicted using prodigal [32]. In the first step, MetaRon identified 822 co-directional proximal gene clusters (IGD < 601 bp), containing 2955 genes. These gene clusters were named as proximons, since they were identified based on direction and intergenic space, as defined by proximon proposition [56–58]. The proximon cluster length range from binary (2 genes) to 32 genes, with no proximons of length 17, 21, 23, 24, 26, 27, 28 and 29 (Fig. 2).

Of the 822 proximal clusters, a third of the clusters demonstrated binary configuration, followed by proximons of length three (19.7%), four (11.8) and greater (35.5%). At this point, it is imperative to highlight that no Transcription Unit Boundary (TUB) is defined in the proximal gene clusters. This means that a proximon might enclose more than one operon or non-operonic genes.

**Fig. 3:**
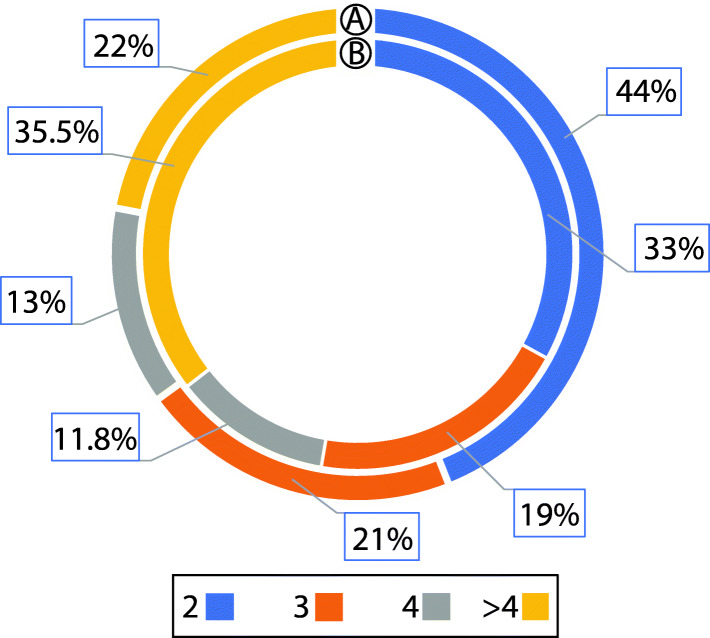
**a** Percentage of *E. coli* K-12 MG1655 operons and (**b**) Percentage of *E. coli* K-12 MG1655 proximons, mapped to one or more reference operons of length 2,3,4 and more than 4 genes

Next, the prediction of promoters further removed the non-operonic genes and clearly defined the transcription unit boundary within the proximons. These filtered proximons are now called operons. The operonic gene clusters contains a promoter upstream of the first and downstream of the last operonic gene. As expected, addition of a stringent structural parameter (promoter) increased the number of operons of length 2,3 and 4 to 364 (43.9%), 176 (21.2%) and 110 (13.2%) operons, respectively. About 21.7% of operons have length ranging between five and sixteen**.** The proportion of operons with length 2–4 increased to 78% as compared to 64.5% of proximon clusters (Fig. 3). The resultant 828 operons contains 2893 genes while, the longest operon is 16 genes long [59–62]. *MetaRon* achieved a sensitivity, specificity and accuracy of 97.8, 94.1 and 92.4%, respectively, when compared with DOOR database [60, 62].

These results corroborate with the fact that most of the operons in *E. coli K12* genome have binary organization [63, 64]. The percentage of binary operons hold a significant importance in accessing the operon predictions since, most of the operons in microbial genomes are binary [14]. An increase in the proportion of such operons in comparison with proximal gene clusters signifies the removal of false positives and improved sensitivity.

#### Simulated genomes

In order to test *MetaRon* with more complex data, we simulated illumine raw reads from whole-genomes of *E. coli* MG1655, *M. tuberculosis* H37Rv and *B. subtilis* 168. The sole reason for this simulation was to create a controlled diversity using genomes belonging to the dominant phyla of the microbiome i.e. *B. subtilis* 168 (*firmicutes), M. tuberculosis* H37Rv (actinobacteria) and *E. coli* MG1655 (proteobacteria) [65]. The simulation of above mentioned 13,266,813 bp long genomes resulted in two million reads simulated at 15X depth via *NeSSM* (Next-Generation Simulator for Metagenomics) [66]. *MetaRon* assembled the simulated reads into 232 scaftigs containing 12,481 genes. Next, 2514 proximons were identified with a gene count of 10,625 genes. The proximons range from 2 to 36 genes in length. In the proceeding step, 2579 operons containing 8749 genes are identified. On comparison with DOOR database *MetaRon* demonstrated the sensitivity, specificity, and accuracy of 93.7, 75.5, and 88.1%, respectively. Since, there is no metagenomic operon prediction method available to draw a comparison. We compared *MetaRon* with MetaProx database, which identified proximons and functional gene clusters from the metagenomic data [56]. The results achieved are encouraging enough to move on to more diverse and complex analysis.

#### *E. coli* C20 draft genome operon prediction

In the third stage of *MetaRon* implementation and performance evaluation, we identified operons from *E. coli* C20 draft genome isolated from the metagenome of chicken gut. MetaRon identified 4544 genes from 4,640,940 bp long genome and resulted in 841 proximons and 946 operons containing 3937 and 2409 genes respectively. The percentage of binary operons significantly increased from 32% (268 proximons) to 71% (673 operons). *MetaRon* achieved a sensitivity, specificity, and accuracy of 87, 91, and 88%, respectively [60, 62].

**Fig. 4:**
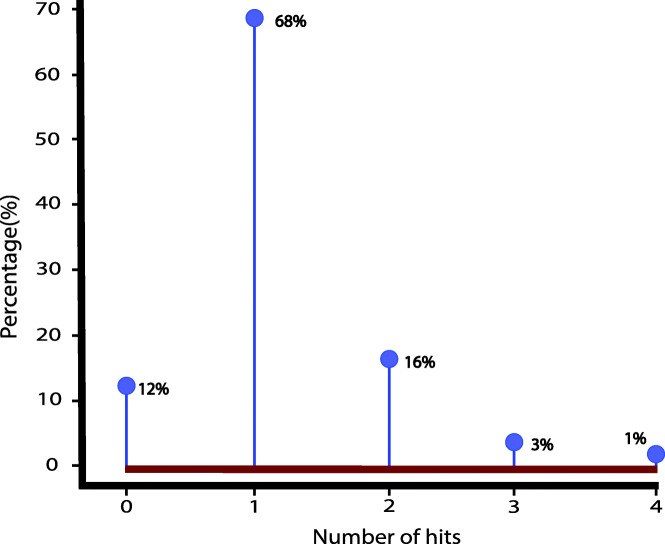
Distribution of *E. coli* C20 operons by the number of hits, when mapped to the reference genome

On comparison with the reference, 68% of the operons discretely mapped to a single reference operon while 20% mapped to more than one operon. Twelve percent of the operons expressed less than 50% identity with the reference hence they were considered as novel or no-hits (Fig. 4). Some variation in the operonic genes could be expected due to the fact that similar genomes could demonstrate variable operonic settings in different conditions [67–70].

**Fig. 5:**
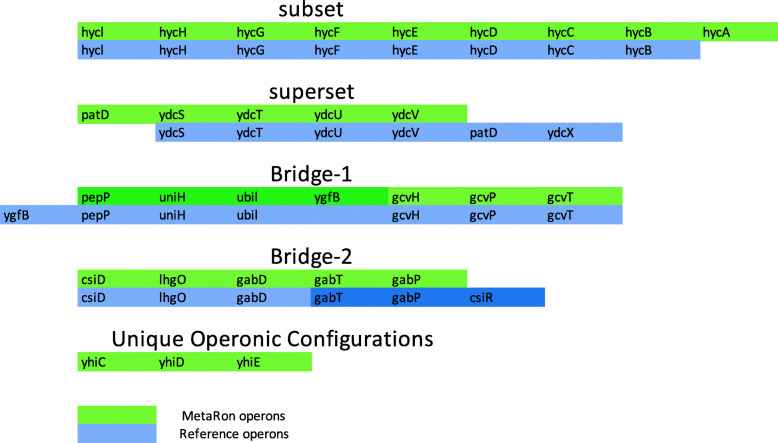
Operonic onfigurations observed when operons predicted by *MetaRon* (green) were mapped to the reference operons (blue)

**Fig. 6:**
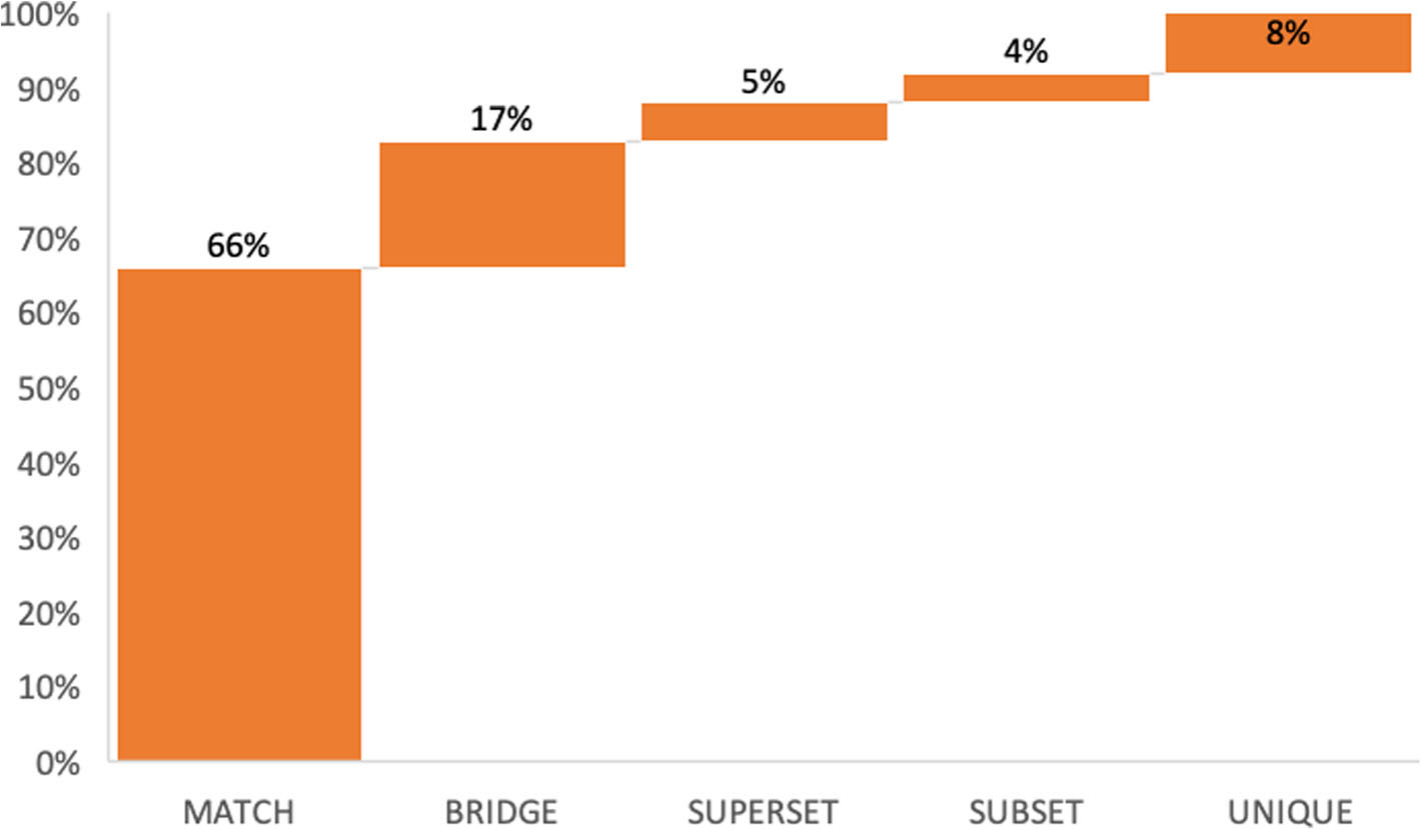
Percentage distribution of various operonic configurations identified by *MetaRon*

Since metagenome data does not have a complete reference, based on which a reference-based-assembly could be performed, De novo assembly usually produces multiple contigs/scaftigs, rather than one long stretch of DNA; hence multiple operonic configurations were observed (Fig. 5). Unlike the proximon proposition, where the majority of the proximons were mapped to more than one operon in a subset fashion, 66% of the operons identified via MetaRon matched precisely to one reference operon as a perfect match. About 8% of the operons show an exact match with one or more extra gene. This is known as a subset (Fig. 5). 4% of the predicted operons displayed contrary formation known as a superset, i.e., the predicted operon contains one or more extra genes as compared to reference operon. (Fig. 6). The subset formations could be due to the distribution of an operon between two scaftigs or different transcription unit boundary (Fig. 5). Furthermore, there were 5% instances when one predicted operon was matched to more than one consecutive operons (bridge-1) or one reference operon was matched to more than one predicted operon (bridge-2). Bridge configurations could be due to altered transcription unit boundary or the inability of the NNPP tool to identify the promoter.

Metagenomic data demonstrates new microbial functions under different levels of stress and environmental stimulus [11]. Many unique operonic organizations are likely to appear as a response to environmental stimuli. This leads to the formation of new or altered operonic configurations such as subsets, supersets or unique operons. In the case of *E. coli* C20, 17% of predicted operons have less than 50% or no match with the reference (Fig. 6). Such unique organizations may well carry precious insights about the microbial activity for a particular environment regarding bacterial products and pathways [11]. Such insights at metagenomic scale could be valuable in understanding disease condition, its prevention and possibly the cure as well.

#### Application to type 2 diabetes metagenomes

**Fig. 7:**
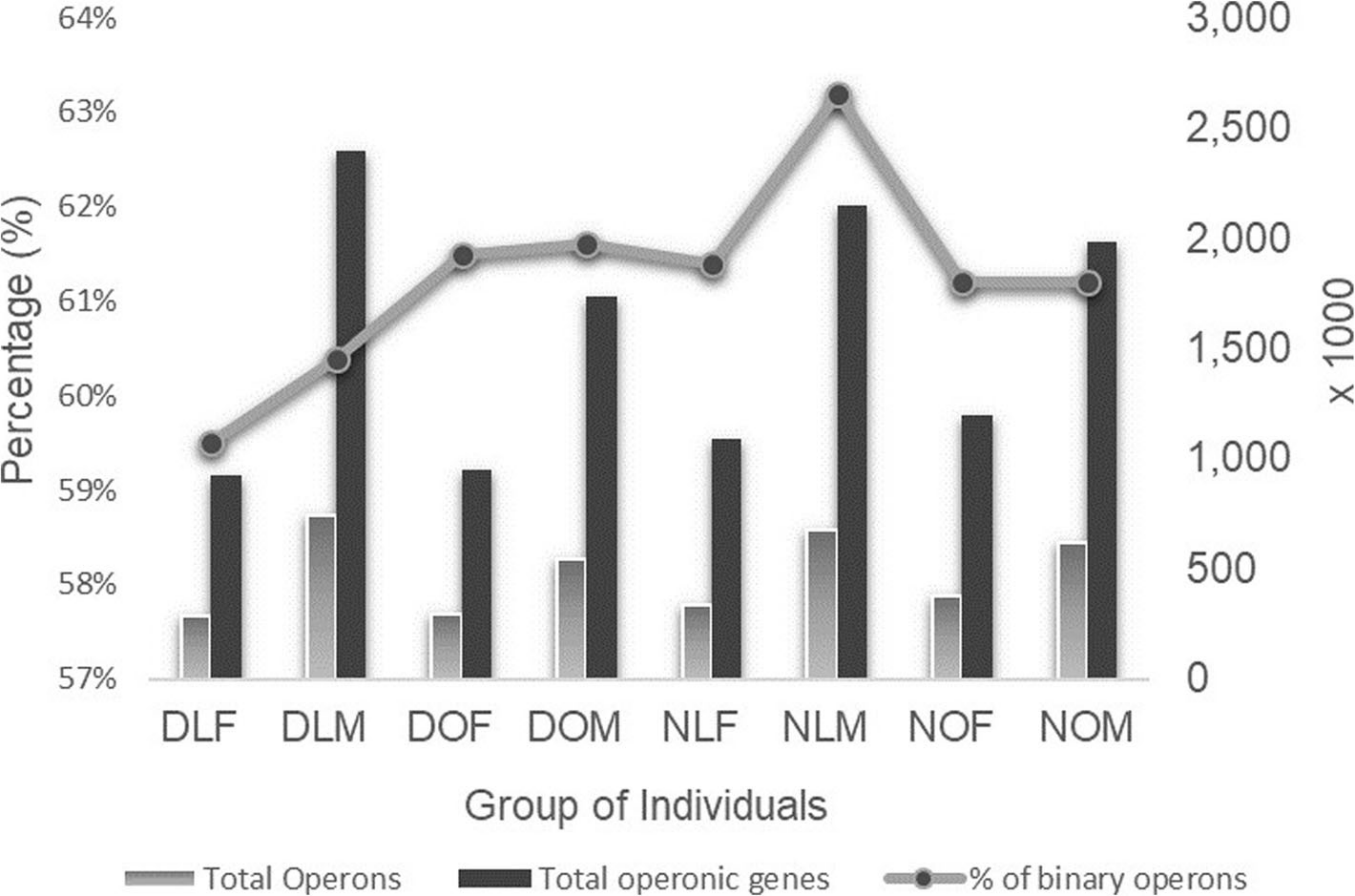
Percentage of binary operons identified in each of the eight group of individuals from metagenomic dataset. The bars also demonstrate the number of metagenomic operons (grey) and operonic genes (black) in each group of individual

*MetaRon* was further implemented on shotgun sequencing reads from the gut of 145 Chinese individuals (74 *Type 2 Diabetic* (T2D), 71 controls) [54]. The two groups of individuals are further divided into four sub-groups in each category based on gender, weight and diabetic/non-diabetic (Table 1). MetaRon identified 3,868,389 operons containing 12,414,125 genes (Fig. 7). This makes up almost 50% of the total 23,280,123 genes. Removing operonic redundancy produced 1.23 million unique operons. The longest operon is 185 genes long. The proportion of binary operons was consistently high in all group of individuals (Fig. 7). On average more than 61% operons had binary setting. The non-redundant set of operon sequences will be used for further analysis including identification of biosynthetic gene clusters and differential pathway analysis.

Technical reasons such as quality of assembly and contig/scaffold length could negatively affect the operon prediction. Furthermore, computational promoter prediction being a tough task might result in missing out some operons. Nevertheless, *MetaRon* performed well at all levels of complexity and the above-mentioned reasons would not undermine the utility of *MetaRon.*

### Prediction of secondary metabolites

**Fig. 8:**
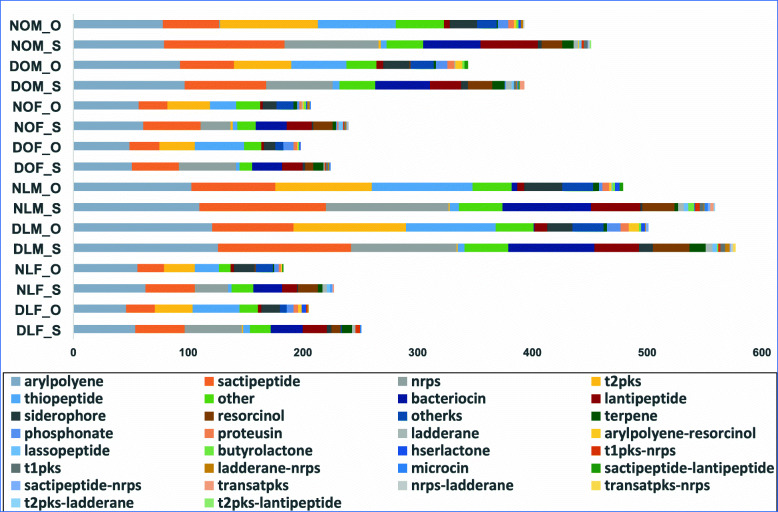
Absolute abundance of Secondary Metabolites predicted from operonic sequences (suffex: “_O”) and whole-metagenomic scaffolds (suffex: “_S”) for each group of individuals from the Metagenomic dataset

**Fig. 9:**
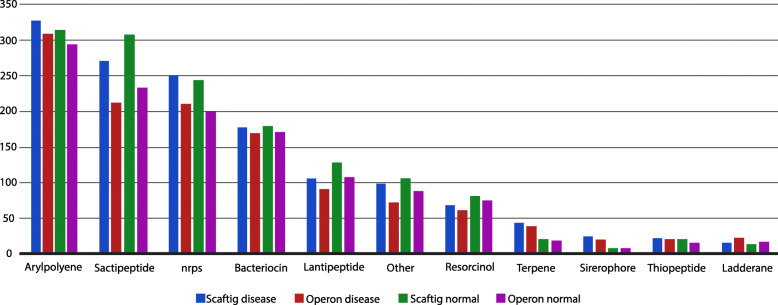
Abundance of Secondary Metabolites predicted from operonic sequences followed the same trends as Secondary Metabolites predicted from whole-metagenomic scaftigs across both disease and controls

**Fig. 10:**
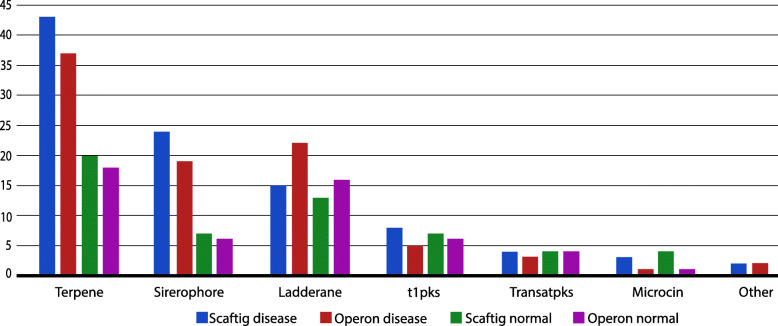
Differentially abundant Secondary Metabolites identified from whole-metagenomic and operonic sequences across both control and disease samples (95% confidence itetrval)

We identified biosynthetic gene clusters (BGCs) from operonic sequences as well as whole-metagenome assembly (Fig. 8). The idea was to demonstrate the association of disease via secondary metabolites (SMs) and also, observe the extent of information operons hold in the metagenomic data. Figure 8 presents a holistic view of the secondary metabolites (SMs) predicted from the operonic sequences and the metagenomic assembly of each group of individuals. As expected, there is a notable change in the abundance of SMs from healthy condition to diabetic state (Fig. 9). Another novel observation is the similar patterns of SMs in operonic sequences and whole-metagenomic assembly (Fig. 9). We normalized the data to test the significance of change in abundance of the secondary metabolites from healthy to disease condition using student’s T-test (95% confidence interval). Several SMs showed significant variance in concentration, as shown in Fig. 10.

### Functional mapping and analysis

Many functional features of the human gut microbiota have shown correlation with health and disease condition. We evaluate the differential abundance of the operonic pathways in association with health and disease condition. The FMAP analysis (See Methodology) was performed between all groups of individuals as mentioned in Table 1. None of the pathways demonstrated differential abundance across all control and disease samples. With exception of *Type 2 Diabetic* lean female (DLF) versus healthy lean female (NLF). No variance in patterns was observed across any group of individuals. The result demonstrates a significant downregulation in several pathways from control to the DLF category of the disease group (*p < 0.01*). To validate if the identified pathways are reported to have association with *type 2* diabetes, we tested and found that most of our findings are consistent with the published literature [71–80]. However, here we also report three pathways to have strong association with *type 2 diabetes*, namely, Maltose phosphorylase (K00691), 3-deoxy-D-glycero-D-galacto-nononate 9-phosphate synthase (K21279) and an uncharacterized protein (K07101). The Maltose phosphorylase catalyzes the phosphorylation process of maltose, resulting in the production of glucose 1-P and glucose. The pathway also overlaps with the glycan degradation [81]. The pathway has never been reported to have any association with T2D, however, glycogen phosphorylase pathway is consistently reported to have strong association with the disease [82, 83]. Further investigation could provide much clear insights into the role of maltose phosphorylase in the occurrence of T2D.

## Conclusion

This study presents a convenient publicly available command line pipeline for the processing of Metagenomic data and operon prediction in shotgun sequencing data. A major advantage of *MetaRon* is that it identifies metagenomic operon independent of any experimental or functional information. MetaRon is therefore the second pipeline that performs systemic identification of metagenomic operons and the first one to do so without any prior functional or experimental information. Considering the complexity and incompleteness of metagenomic data, the pipeline predicts metagenomic operons with very high specificity. This study is also one of the first attempts to perform a detailed downstream analysis of the metagenomic operons and explaining the occurrence of the disease from the operonic point of view.

The differential abundance of operonic secondary metabolites and pathways demonstrated the same trend as of whole metagenome, thus highlighting the amount of information carried by the operons. It also suggests that for the association of secondary metabolites with disease/healthy condition, operons could also act as a subset to represent the whole-metagenomic sample. *MetaRon* promises to be a useful pipeline in the identification of operons from whole-genome and metagenome shotgun sequencing data. It is quite certain that more in-depth investigation, aided with wet-lab resources, could provide insightful findings about the diverse microbial biosphere. In this research, the analysis was performed separately on the *MetaRon* predicted operons, however, in the future we plan to integrate the prediction of secondary metabolites, pathway annotation and graphical representation within the pipeline.

## Availability and requirements

**Project name:** *MetaRon* (Metagenomic opeRon Prediction pipeline).

**Project Source code availability:** https://github.com/zaidissa/MetaRon

**Operating system:** Linux.

**Programming language:** Python > 3.0.

**License:** BSD License.

**Any restriction to use by non-academics:** Academic use only.

**Contact:** syedzaidi85@hotmail.co.uk, drimran@zju.edu.cn

## Data Availability

All data-sets used in the development, testing and analysis of MetaRon are publicly available as described in the methods section. Using the tutorial code, user can reproduce the results provided at https://github.com/zaidissa/MetaRon. MetaRon is designed for Linux. The installation instructions are covered in detail at https://github.com/zaidissa/MetaRon
